# Lipoxin and Aspirin-Triggered Lipoxins

**DOI:** 10.1100/tsw.2010.113

**Published:** 2010-06-02

**Authors:** Mario Romano

**Affiliations:** Department of Biomedical Sciences, Aging Research Center, Ce.S.I., “Gabriele D'Annunzio” University Foundation, Chieti, Italy

**Keywords:** arachidonic acid, lipoxin, lipoxygenase, cyclooxygenase, inflammation, aspirin, receptor

## Abstract

Lipoxins and their 15 epimers, aspirin triggered lipoxins (ATL), are eicosanoids derived from sequential lipoxygenase (LO) metabolism of arachidonic acid. The main routes of lipoxin biosynthesis involve cooperation between 15- and 5-LO, and between 12- and 5-LO. ATL are generated by interactions between 5-LO and aspirin-acetylated cyclooxygenase-2. Cellular models recapitulating these interactions involve leukocytes, platelets, vascular endothelium, and epithelium. To circumvent rapid lipoxin and ATL metabolism and inactivation, stable analogs, bearing potent and long-lasting biological activity, have been synthesized. Some of these analogs displayed therapeutic potential by showing strong anti-inflammatory activity in a number of animal models of disease, including reperfusion injury; arthritis; gastrointestinal, renal, respiratory, and vascular inflammatory disorders; eye damage; periodontitis; and selected infectious diseases. Counter-regulatory signaling by lipoxin A_4_ and 15-epi-lipoxin A_4_ is triggered by the activation of a seven-transmembrane domain receptor, termed FPR2/ALX, which is highly expressed in myeloid cells and has been recognized as a main anti-inflammatory receptor.

### STRUCTURE

The presence of polar compounds carrying a conjugated tetraene chromophore was first revealed in incubations of human leukocytes with arachidonic acid or 15*S*-hydroperoxy-5,8,11,13-eicosatetraenoic acid (HpETE)[1,2]. Structure elucidation of the compounds formed during these incubations revealed 5*S*,6*R*,15*S*-trihydroxy-7,9,13-*trans*-11-*cis*-eicosatetraenoic acid and 5*S*,14*R*,15*S*-trihydroxy-6,10,12-*trans*-8-*cis*-eicosatetraenoic acid, which were termed, respectively, lipoxin $A_4$ and lipoxin $B_4$ (Fig. 1), where lipoxin stands for **lipox**ygenase **in**teraction products. The UV absorption spectrum of both lipoxin $A_4$ and $B_4$ shows pre-eminent absorbance bands at $\lambda^{MeOH}_{max}$ 287, 300, and 315 nm, with a weaker band at 270 nm and a molar extinction coefficient of 50,000 $M^{-1}$/cm. Monitored using negative ion mode mass spectrometry with electrospray ionization, lipoxins yield [M-H]- parent ions of 351 m/z and diagnostic MS/MS product ions at *m/z* ***333*** [351-$H_2O$], ***315*** [351-2$H_2O$], ***307*** [351-$CO_2$], ***289*** [351-$H_2O$-$CO_2$], ***271*** [351-2$H_2O$-$CO_2$], ***251*** [351-CHO$(CH_2)_4CH_3$], ***235*** [351-CHO$(CH_2)_3COO^-$], ***233*** [351-$H_2O$-CHO$(CH_2)_4CH_3$], ***219*** [351-CHO$(CH_2)_3COO^-$-O], ***207*** [351-$CO_2$-CHO$(CH_2)_4CH_3$], ***189*** [351-$H_2O$-$CO_2$-

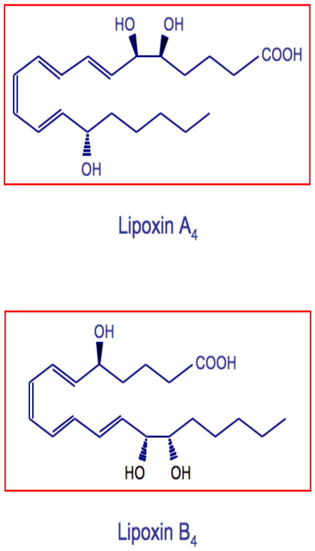

**FIGURE 1.** Chemical structures of lipoxin $A_4$ and $B_4$.

$CHO(CH_2)_4CH_3$], ***135*** [351-$CHO(CH_2)_3$-COOH-$CHO(CH_2)_4CH_3$], ***115*** [$CHO(CH_2)_3COO^-$] for lipoxin $A_4$, and *m/z* ***333*** [351-$H_2O$], ***315*** [351-2$H_2O$], ***307*** [351-$CO_2$], ***289*** [351-$H_2O$-$CO_2$], ***271*** [351-2$H_2O$-$CO_2$], ***251*** [351-$CHO(CH_2)_4CH_3$], ***233*** [351-$H_2O$-$CHO(CH_2)_4CH_3$], ***221*** [351-$CHOCHOH(CH_2)_4CH_3$], ***207*** [351-$CO_2$-$CHO(CH_2)_4CH_3$], ***189*** [351-$H_2O$-$CO_2$-$CHO(CH_2)_4CH_3$], ***163*** [351-$CO_2$-$CH_2COHCHOH(CH_2)_4CH_3$], ***129*** [$CH_3CO(CH_2)_3COO^-$] and ***115*** [$CHO(CH_2)_3COO^-$] for lipoxin $B_4$.

Although a number of lipoxin $A_4$ and $B_4$ isomers have been identified *in vitro*, their biological significance remains incompletely defined[3,4].

### BIOSYNTHESIS

Lipoxins and ATL are generated by cooperation between lipoxygenase isoforms and aspirin-acetylated cyclooxygenase-2 (COX-2) and 5-lipoxygenase (5-LO), respectively. Two main routes of lipoxin biosynthesis have been characterized: the 5-/15-LO and the 5-/12-LO.

#### The 5-/15-LO Pathway

This pathway can be alternatively initiated by 5- or 15-LO. In one case, 5-LO converts arachidonic acid to leukotriene (LT) $A_4$, which is further metabolized by 15-LO to a 5*S*,6*S*,15*S*-epoxytetraene intermediate, enzymatically transformed into lipoxin $A_4$ and $B_4$. In the other event, arachidonic acid is converted by 15-LO into HpETE, which is reduced to 15*S*-hydroxy eicosatetreanoic acid (HETE) by a peroxidase. 15*S*-HpETE and 15*S*-HETE are transformed by 5-LO into the 5*S*,6*S*,15*S*-epoxytetraene intermediate, which yields both lipoxin $A_4$ and $B_4$[5]. Cellular models for this biosynthetic route are human polymorphonuclear neutrophils (PMNs) (Fig. 2A)[6], eosinophils[7], alveolar macrophages[8], or interactions between PMNs and eosinophils[7], or PMNs and lung tissue (Fig. 2B)[9]. The 5-/15-LO pathway appears to be predominant in the respiratory tract and it may be relevant within the brain, since lipoxins are generated during cocultures of HIV-infected monocytes and astroglia[10]. *In vivo* evidence of 15-LO–dependent lipoxin biosynthesis was obtained in a rat model by transfection of the human 15-LO gene into one of the kidneys. The transfected kidney produced higher amounts of urinary immunoreactive lipoxin $A_4$ compared to the untransfected kidney[11].

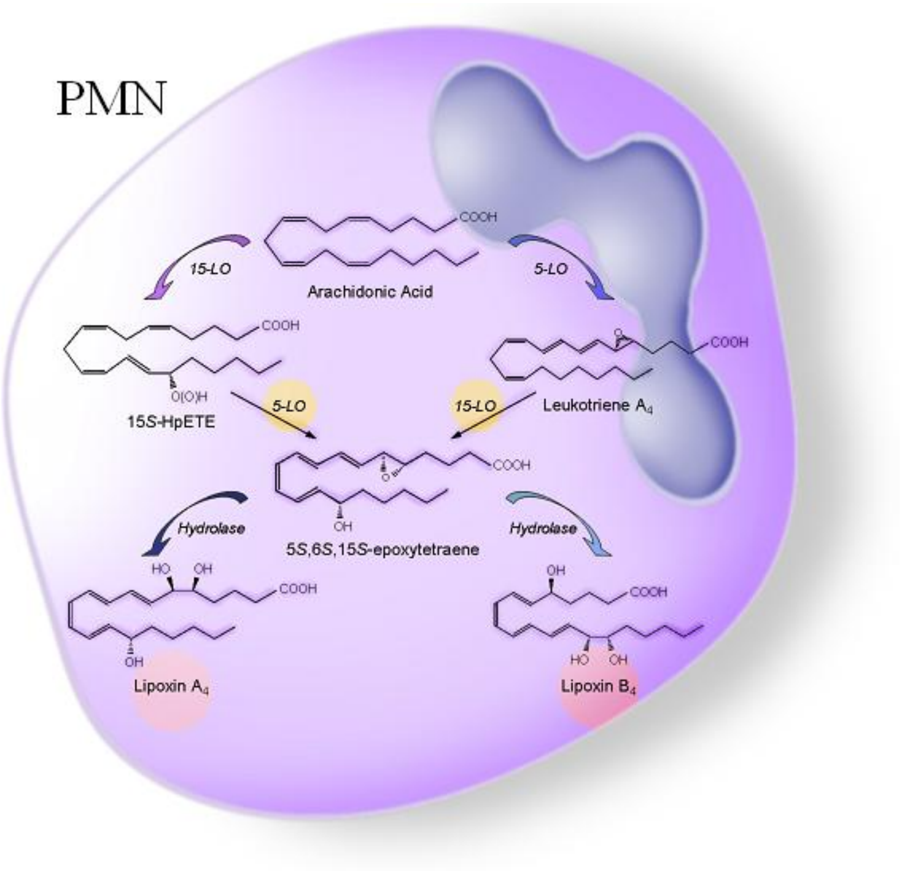

**FIGURE 2A.** The 5-/15-LO pathway of lipoxin biosynthesis in PMNs.

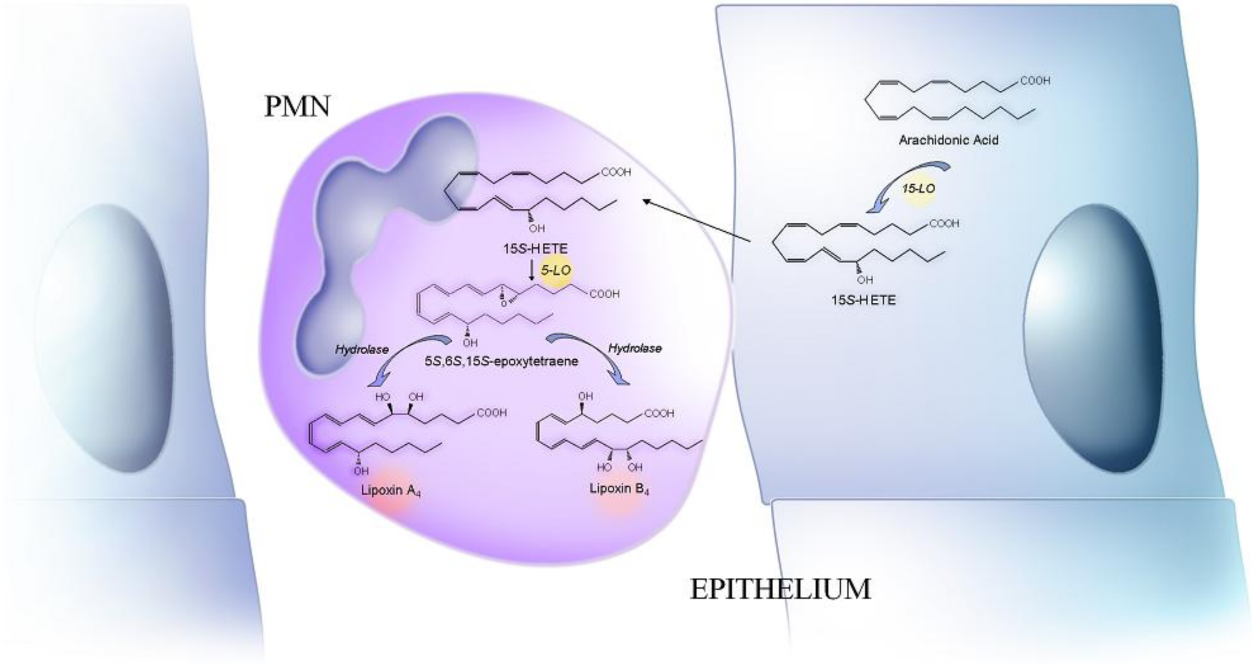

**FIGURE 2B.** The 5-/15-LO pathway of lipoxin biosynthesis during interactions between PMNs and epithelial cells.

### The 5-/12-LO Pathway

This pathway was initially characterized in mixed PMN-platelet incubations[12,13,14] and then further elucidated with megakaryocytes[15], 12-LO–transfected cells[15], platelets exposed to leukotriene $A_4$ ($LTA_4$)[16], and human platelet recombinant 12-LO incubated with $LTA_4$ in a cell-free system[17]. In this system, 12-LO converted $LTA_4$ into lipoxin $A_4$ and $B_4$ with an apparent $K_m$ of 7.9 ± 0.8 μM, whereas the calculated $V_{max}$ was 24.5 ± 2.5 nmol/min/mg of protein[17]. Notably, recombinant 12-LO showed a comparable affinity for arachidonic acid, its main substrate ($K_m$ of 6.2 ± 1.8 μM)[17], suggesting that the lipoxin synthase activity of this enzyme is of primarily biological meaning. In order to generate lipoxins, 12-LO converts $LTA_4$ into a delocalized cation by proton transfer, following hydrogen abstraction from carbon-13 to insert molecular oxygen at carbon-15. The cation is attacked by water at carbon-6 to give lipoxin $A_4$ and at carbon-14 to yield lipoxin $B_4$[15] (Fig. 3). 12-LO–governed biosynthesis of lipoxin $B_4$, but not of lipoxin $A_4$, is regulated by mechanism-based inactivation[16,17], suggesting that the 12-LO attack at carbon-14 of the delocalized cation makes the lipoxin $B_4$ formation site inaccessible for further substrate cycling.

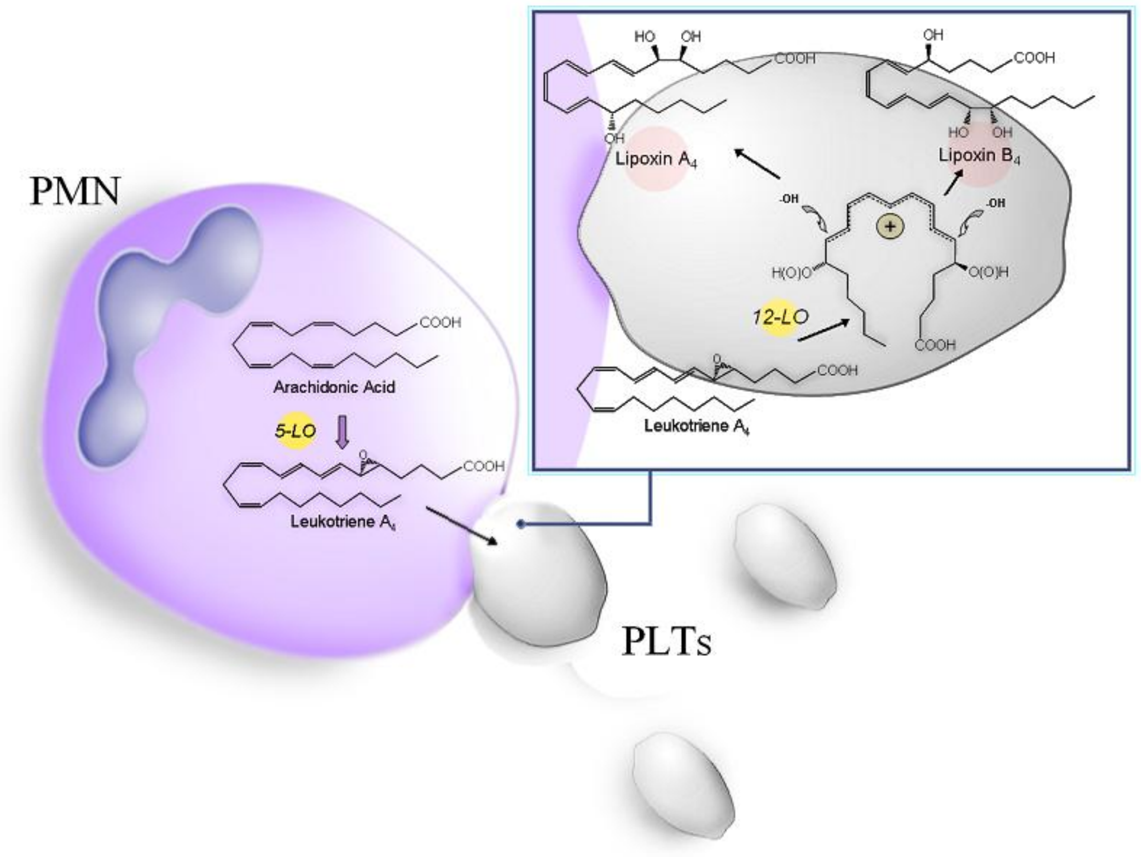

**FIGURE 3.** The 5-/12-LO pathway of lipoxin biosynthesis during interactions between PMNs and platelets.

*In vivo* evidence of the 5-/12-LO route of lipoxin generation has been obtained in patients with cardiovascular disease, during coronary angioplasty[18], and in healthy subjects undergoing strenuous physical exercise[19]. In both conditions, enhanced transcellular exchanges between PMNs and platelets have been documented. This route also occurs in trout macrophages to generate lipoxin $A_4$[20], confirming the evolutionary origin of lipoxins.

### The Origin of the 15-Epi-Lipoxins

The formation of epi-lipoxins was first detected in mixed incubations of aspirin-treated human umbilical endothelial cells with PMNs[21], which were done on the basis of previous observation that aspirin did not completely suppress COX-2 catalytic activity, but rather redirected it to the transformation of arachidonic acid into 15*R*-HETE[22]. Among the number of tetraenes formed during these incubations, 15-epi-lipoxin $A_4$ and 15-epi-lipoxin $B_4$ were identified by UV spectroscopy and mass spectrometry. Fifteen-epi-lipoxins were therefore collectively termed aspirin-triggered lipoxins (ATL). Their biosynthesis proceeds through the conversion of 15*R*-HETE by 5-LO to an epoxide intermediate, which, similarly to what occurs during lipoxin formation by 5-/15-LO interactions, is enzymatically converted into 15-epi-lipoxin $A_4$ (ATLa) and 15-epi-lipoxin $B_4$ (Fig. 4). Consistently, ATL were formed by $A_{23187}$-stimulated PMN exposed to 15*R*-HETE[21]. Additional cellular models of ATL formation have been reported, including interactions between PMNs and A459 cells[23], or between hepatocytes and liver cells[24]. In accordance, ATL formation was observed in liver tissue from aspirin-treated rats[24]. ATL and lipoxins can also be formed upon stimulation of whole blood *ex vivo*[25,26] and their amount is increased in aspirin-tolerant asthmatics compared to aspirin-intolerant asthmatics[26]. Recently, the first evidence of ATL formation in healthy volunteers taking aspirin has been obtained[27]. This was confirmed in a larger randomized clinical trial[28]. Additional circuits of ATL biosynthesis have been unveiled. One is regulated by cytocrome *P*-450, which can be stimulated by aspirin and forms 15*R*-HETE, as intermediate[24]. Another appears to be triggered by pioglitazone and atorvastatin in the rat[29], or by lovastatin via 14,15-epoxyeicosatrienoic acid (14,15-EET) generation[30].

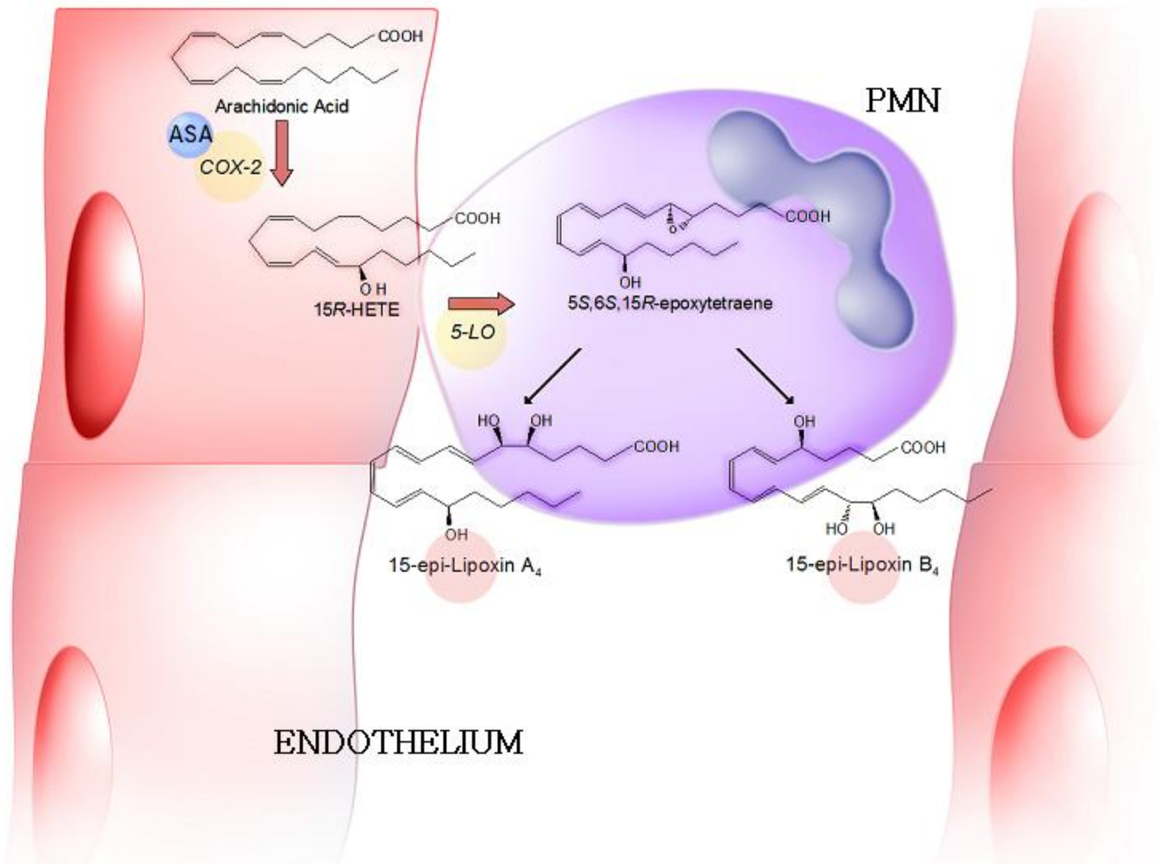

**FIGURE 4.** ATL biosynthetic pathway during interactions between aspirin-treated endothelial cells and PMNs.

## BIOACTIONS

### The Stable Analogs

Since their first identification, lipoxins and ATL have displayed an array of biological actions consistent with anti-inflammatory, proresolution profiles. In particular, they block chemotaxis, adherence to microvasculature, transendothelial and transepithelial migration of PMNs[31,32,33,34], and chemotaxis of eosinophils[35,36], while promoting monocyte chemotaxis and nonphlogistic phagocytosis of apoptotic PMNs by macrophages[37,38,39]. In addition, lipoxin $A_4$ and ATLa limit peroxynitrite formation and NF-κB activation in leukocytes[40], PMN azurophilic degranulation[41], the release of the proinflammatory cytokines IL-6 and IL-8[42], TNF-α–induced superoxide anion generation, and IL-1β release by PMNs[43], while they stimulate the release of the antiphlogistic cytokine TGF-β in mice[44]. Antiangiogenic and antifibrotic activities of lipoxin $A_4$ and ATLa have also been reported[45,46,47,48,49].

A main limitation for *in vivo* studies with native lipoxins is represented by their short half-life, since they undergo rapid metabolic inactivation. In the case of lipoxin $A_4$, the main metabolic pathways occur in monocyte/macrophages and involve initial dehydrogenation to 15-oxo-lipoxin $A_4$ by 15-hydroxyprostaglandin dehydrogenase (15-PGDH)[50]. The 15-oxo-lipoxin $A_4$ is converted by the 15-oxoprostaglandin 13-reductase to 13,14-dihydro-15-oxo-lipoxin $A_4$, which is further metabolized by 15-PGDH to yield 13-14-dihydro-lipoxin $A_4$[51]. Lipoxin $B_4$ undergoes a similar metabolic sequence[52]. $\omega$-Hydroxylation and $\omega$-oxidation pathways have been also reported[53,54,55]. Metabolic inactivation of ATL has been also observed, although 15-epi-lipoxin$A_4$ appears to be more resistant than lipoxin $A_4$ to dehydrogenation[31].

Because of lipoxin and ATL metabolic inactivation, a number of stable analogs have been synthesized in recent years, mainly focusing on the A series of lipoxins. The first series were designed to minimize 15-PGDH and $\omega$-oxidation–dependent inactivation. Among these were 15*R/S*-methyl-lipoxin $A_4$ and 16-phenoxy-lipoxin $A_4$[31]. These analogs retained receptor-binding affinity and full biological activity in assays of neutrophil transmigration across intestinal epithelial cells[31]. Further improvement in activity and stability was achieved by adding a fluoride to the phenoxy ring of 16-phenoxy- and 15-epi-16-phenoxy-lipoxin $A_4$ to yield, respectively, 16-(parafluoro)-phenoxy-lipoxin $A_4$ and 15-epi-16-(parafluoro)-phenoxy-lipoxin $A_4$[56]. Recently, 3-oxa derivatives of 15-epi-16-(parafluoro)-phenoxy-lipoxin $A_4$ have been synthesized, showing marked resistance to β-oxidation[57]. More recently, aromatic, pyridin, and benzo analogs have been obtained with enhanced anti-inflammatory properties[58,59,60]. The analogs have been largely used to assess the pathophysiological relevance of lipoxins and ATL *in vivo*. The large majority of these studies were conducted with lipoxin $A_4$ and ATLa derivatives and showed consistent anti-inflammatory, proresolution properties in a variety of animal models. Although these models have a number of built-in limitations related to species specificity of responses, to the administration of lipoxin $A_4$ prior to the exposure to the pathological agent, with the exception of a very recent study showing proresolution properties of ATLa administered to mice bearing peritonitis[61], and to the modality of disease induction, nevertheless they provide useful indications for future use of lipoxin $A_4$ and ATLa in human disease. The best-characterized models of disease that may benefit from treatment with lipoxin $A_4$ and ATLa are listed below.

## DISEASE MODELS

### Respiratory Tract

#### *Asthma and Allergic Diseases*

Initial studies in asthmatics showed that inhaled native lipoxin $A_4$ attenuated $LTC_4$–induced airway obstruction[62]. It was later demonstrated that lipoxin $A_4$ and ATLa inhibited allergen-induced eosinophilic pleurisy in sensitized rats, with a mechanism involving IL-5 and eotaxin production[36]. More recent studies showed bronchodilatory, anti-inflammatory effects of lipoxin $A_4$ and ATLa analogs. For example, ATLa and 3-oxa-15-epi-lipoxin $A_4$ reduced leukocyte recruitment, cysteinyl LTs, IL-4, IL-10, and IL-13 in the lung of sensitized mice challenged with ovalbumin[63]. In another study, a lipoxin $A_4$ analog reduced airway hyper-responsiveness, airway inflammation, and eosinophil infiltration, as well as IL-5 and eotaxin levels[64]. Of note, lipoxin $A_4$ may play a key role in the homeostasis of airway epithelium since it up-regulates the expression of the tight junction proteins zonula occludens-1, claudin-1, and occludin, sustaining transepithelial electrical resistance[65]. Thus, lipoxin $A_4$ may also promote epithelial repair in the airway.

Consistent with the protective action of endogenous lipoxins and ATL, patients with asthma or allergic rhinitis display reduced levels and biosynthetic potential of these eicosanoids[66].

#### *Acute Lung Injury*

Evidence is accumulating of lipoxin $A_4$ and ATLa protection of acid-induced acute lung injury. Mechanisms are related to the modulation of the expression of the lipoxin $A_4$ receptor, the inhibition of the release of inflammatory cytokines, and neutrophil apoptosis[67,68]. In addition, benzolipoxin analogs protected lungs from hindlimb ischemia-reperfusion injury of the lung[60]. In another study, 15-epi-16-(parafluoro)-phenoxy lipoxin $A_4$ protected the mice lung from LPS-induced acute lung injury, with a heme-oxygenase-1–dependent mechanism[69]. Finally, ATLa prevented inflammatory and fibrotic reactions in bleomycin-induced pulmonary fibrosis, improving pulmonary mechanics and survival[49].

#### *Cystic Fibrosis*

Neutrophilic lung disease is a trademark of cystic fibrosis (CF). Reduced levels of lipoxin $A_4$ in bronchoalveolar lavage fluid from CF patients was recently detected[70], suggesting that the sustained inflammatory response in CF may be related to impairments of resolution pathways. Consistently, 15-epi-16-(parafluoro)-phenoxy-lipoxin $A_4$ blocked *Pseudomonas aeruginosa*–induced IL-8 secretion and PMN recruitment in mice[70]. This ATLa analog also ameliorated disease progression in mice exposed to *P. aeruginosa*[70]. Notably, antibiotics increased lipoxin $A_4$ and decreased IL-8 levels in CF sputum[71].

### Joints

#### *Rheumatoid Arthritis*

The observation that lipoxin $A_4$ inhibits proinflammatory responses of human synovial fibroblasts, i.e., matrix metalloproteinase and cytokine release[42], suggests that lipoxins and ATL may be beneficial in rheumatoid arthritis (RA). Synovial tissues from RA patients exhibited enhanced expression of the lipoxin $A_4$ receptor compared to patients with osteoarthritis[72]. Likewise, synovial fluids from RA patients showed higher concentrations of lipoxin $A_4$ and ATL[72]. Thus, up-regulation of the lipoxin $A_4$/lipoxin $A_4$ receptor dyad appears to represent a response to injury mechanism in RA. Whether it may convey anti-inflammatory signaling remains to be determined. The relevance of the lipoxin $A_4$ receptor within the context of RA is underscored by the recent observation that BML-111, a lipoxin $A_4$ receptor agonist, reduced disease activity scores and joint destruction in a collagen-induced arthritis mouse model[73]. Although further evidence is needed, collectively, these results provide the background for the potential use of lipoxin $A_4$ and ATLa derivatives in RA.

## Gastrointestinal Apparatus

### *Stomach*

Gastrolesivity represents a main drawback to chronic administration of aspirin. ATL biosynthesis by the rat gastric mucosa following aspirin administration has been reported[74], suggesting that ATL formation by aspirin-acetylated COX-2 may represent a mechanism of gastric adaptation to aspirin. Thus, it may be reasoned that aspirin-related gastric damage occurs when the buffering capability of ATL becomes insufficient. Indeed, lipoxin $A_4$ protected the rat gastric mucosa from aspirin-induced damage[75]. In healthy humans, aspirin enhanced ATLa urinary levels, which were suppressed by selective COX-2 inhibitors (Coxibs)[27], underlying the potential increased risk of gastric damage when aspirin is coadministered with Coxibs. However, in a more recent study with a cohort of 24 patients affected by ischemic heart disease in association with osteoarthritis, chronically treated with aspirin, the administration of selective or nonselective COX inhibitors did not significantly change urinary ATL levels[76], indicating that in this specific clinical setting, other factors may influence ATL excretion. On the other hand, evaluation of gastric ATLa production may be more informative regarding the protective role of this eicosanoid in protection from gastric damage induced by COX inhibitors, since urinary ATLa levels do not necessarily reflect ATLa biosynthesis within the gastric mucosa. Lipoxin $A_4$ protection of gastric damage induced by ethanol, sodium salicylate, or ischemia reperfusion has been reported in rats[77,78], extending the array of etiopathogenetic events that can benefit from lipoxin $A_4$ or ATLa administration.

### *Bowel*

An early study showed that lipoxin $A_4$ limited neutrophil transmigration across the intestinal epithelium[32] and protected, as well as ATLa, colonocytes from TNF-α–induced apoptosis[79], suggesting that lipoxins and ATL may be protective during intestinal inflammatory disorders. This hypothesis was challenged and proved to be correct in a number of studies. Lipoxin analogs attenuated dextran sodium sulfate–induced colitis[80]. β-Oxidation–resistant lipoxin $A_4$ analogs ameliorated hapten-induced colitis[81]. These effects are likely to be receptor mediated, since the intestinal epithelium expresses the lipoxin $A_4$ receptor[82], which is preferentially localized on the basolateral surface of polarized cells[83]. Remarkably, ATLa showed significant proresolution activity when administered to mice bearing peritonitis[61].

Lipoxin $A_4$ inhibition of NF-κB activation in intestinal epithelial cells has been recently reported[84], providing further insight into the molecular mechanism of lipoxin $A_4$ protective action in gastrointestinal inflammatory disorders.

## Urinary Tract

The involvement of lipoxins and ATL in renal pathophysiology has been established by a number of studies *in vitro* and *in vivo*. In mesangial cells, lipoxin $A_4$ antagonized a number of proinflammatory, hemodynamic, fibrotic responses, such as LT-dependent decrease in filtration rate[85] and neutrophil adhesion[86]; PDGF-induced proliferation[87,88], via inhibition of Akt/PKB signaling[89] and profibrotic gene expression[48]; and connective tissue growth factor–stimulated chemokine production[90]. *In vivo*, 15-epi-16-(parafluoro)-phenoxy-lipoxin $A_4$ displayed protective properties in a rat model of ischemic acute renal failure[91]. Consistently, transcriptomic analysis during murine ischemia-reperfusion injury revealed that this analog regulated the expression of a number of cytokines, growth factors, adhesion molecules, and proteases, with a renoprotective profile[92].

Further support to the renoprotective activity of lipoxin $A_4$ and ATLa is provided by the observation of lipoxin $A_4$ biosynthesis and function maintenance following rat kidney transfection with 15-LO in experimental antibody-induced glomerulonephritis[11].

### Eye

Endogenous generation of lipoxin $A_4$ by mouse corneas has been recently observed[93]. Topical treatment with lipoxin $A_4$ promoted corneal re-epithelialization and attenuated thermal injury[94], with a mechanism involving heme-oxygenase-1 up-regulation[95]. In a rat model of endotoxin-induced uveitis, topical lipoxin $A_4$ reduced the inflammatory infiltrate and the protein leakage into the aqueous humor. It also inhibited accumulation of IL-1β and TNF-α, as well as NF-κB and c-Jun activation[96].

ATLa attenuated suture- or micropellet-induced corneal neovascularization in mice, reducing neutrophil and macrophage and lowering mRNA levels of TNF-α, IL-1α, IL-1β, VEGF-A, VEGF-C, and VEGFR2[97]. Consistently, topical lipoxin $A_4$ rescued 15-LO knockout mice from suture-induced exacerbated angiogenesis[98].

Together, these results provide substantial support to therapeutic use of lipoxin $A_4$ and ATL analogs in selected diseases of the eye.

### Vascular Disease

Lipoxins regulate key pathways of vascular homeostasis. Early studies showed lipoxin stimulation of prostacyclin secretion by endothelial cells[99] and vasorelaxant effects[100], indicating that lipoxins may regulate the vascular tone. The regulatory effect of ATLa on nitric oxide release was also reported[101], although no evidence of direct lipoxin impact on NO biosynthesis has yet been presented. Consistent with a vasoprotective profile, ATLa inhibited generation of reactive oxygen species by endothelial cells[102]. Best characterized is the impact of lipoxins and ATL on angiogenic pathways. Modulation of endothelial cell proliferation, as well as VEGF and VEGF receptor expression by lipoxin $A_4$ and ATLa, have been described in a variety of experimental settings as part of an anti-inflammatory response[45,46,97,103,104].

Recently, the hypothesis that endogenous anti-inflammatory, proresolution circuits, including lipoxin and ATL biosynthesis, may be altered in atherosclerosis has been put forward[105]. This model of disease, as well as vasculitis and other degenerative vascular disease, may represent interesting fields of investigation of lipoxin and ATL functions within the context of vascular pathobiology.

### Infectious Disease

#### *Parasite*

Lipoxin $A_4$ appears to regulate IL-12 generation by dendritic cells exposed to a *Toxoplasma gondii* extract[106]. Induction of lipoxin $A_4$ biosynthesis by *T. gondii in vivo* has been reported[107]. This appears to represent a host defense mechanism, since the administration of 15-epi-16-(parafluoro)-phenoxy-lipoxin $A_4$ prevented postinfection mortality of 5-LO knockout mice[106]. Consistently, plant 15-LO generated endogenous lipoxin $A_4$ and suppressed *T. gondii*–induced production of IL-12 by splenic dendritic cells[108].

#### *TBC*

Mice infected with *Mycobacterium tuberculosis* produced high levels of lipoxin $A_4$, which were substantially reduced in 5-LO knockout animals[109]. Administration to these mice of 15-epi-16-

(parafluoro)-phenoxy-lipoxin $A_4$ significantly reduced bacterial growth in the lung as well as IFN-γ production by splenocytes. Macrophages are likely to represent a main source of lipoxin $A_4$ upon *M. tuberculosis* infection[110].

#### *Periodontitis*

Lipoxin $A_4$ analogs inhibited leukocyte recruitment elicited by *Phorphyromonas gingivalis* in a murine air pouch model[111]. Remarkably, topical application of 15-epi-16-(parafluoro)-phenoxy-lipoxin $A_4$ drastically reduced leukocyte infiltration, bone loss, and inflammation in a rabbit model of acute periodontitis[112].

#### Reperfusion Injury

Lipoxin and ATL protection from reperfusion injury is not restricted to the renal district. In fact, ATLa inhibited PMN infiltrates in the lung following hindlimb ischemia reperfusion[113], suggesting that lipoxins and ATL may antagonize stress responses, such as those observed in perioperative medicine. Along these lines, lipoxin $A_4$ and ATLa reduced the expression of the ischemia-induced chemokine and cytokine-induced neutrophil chemoattractant (CINC)-1 in rat liver[114].

Furthermore, in a rat model of transient focal cerebral ischemia, induced by middle cerebral artery occlusion, intracerebroventricular administration of a lipoxin $A_4$ analog reduced infarction volume and improved neurological dysfunctions[115]. Down-regulation of proinflammatory cytokines TNF-α and IL-1β, as well as of NF-κB, and up-regulation of anti-inflammatory cytokines IL-10 and TGF-$\beta_1$ in the ischemic brain were observed[116]. The lipoxin $A_4$-IL-10 axis seems to play a relevant role in protection from reperfusion injury, since lipoxin $A_4$ failed to reduce inflammation and tissue damage in IL-10–deficient mice[116].

A schematic representation of diseases that may benefit from treatment with lipoxin $A_4$ and ATLa is reported in Fig. 5.

### FPR2/ALX, THE LIPOXIN $A_4$ RECEPTOR

Lipoxin $A_4$, ATLa, and analogs anti-inflammatory functions are achieved through the activation of a specific receptor, spanning seven-transmembrane domains and belonging to the family of chemotactic receptors[117,118]. This receptor, initially termed FPRL1, has been recently renamed FPR2/ALX[119]. A comprehensive description of FPR2/ALX characteristics is beyond the scope of this review. Nevertheless, it is worth mentioning that this receptor is highly expressed in cells of the immune inflammatory response, i.e., PMNs, monocytes, lymphocytes, and endothelial cells, and that in addition to lipoxin $A_4$, ATLa, and their stable analogs, it is recognized by a number of peptides, including the anti-inflammatory annexin A1 and the proinflammatory serum amyloid A (reviewed in Romano et al.[120]). Notably, the potent anti-inflammatory resolvin D1 is a partial agonist for this receptor[121], suggesting complex interactions among anti-inflammatory receptors and their agonists. Studies with transgenics overexpressing Fpr2, the mouse hortholog of FPR2/ALX in myeloid cells or with Fpr2 knocked out[122,123], conclusively proved the predominant anti-inflammatory properties of this receptor, which thus may represent a potential pharmacological target for treatment of inflammatory disorders.

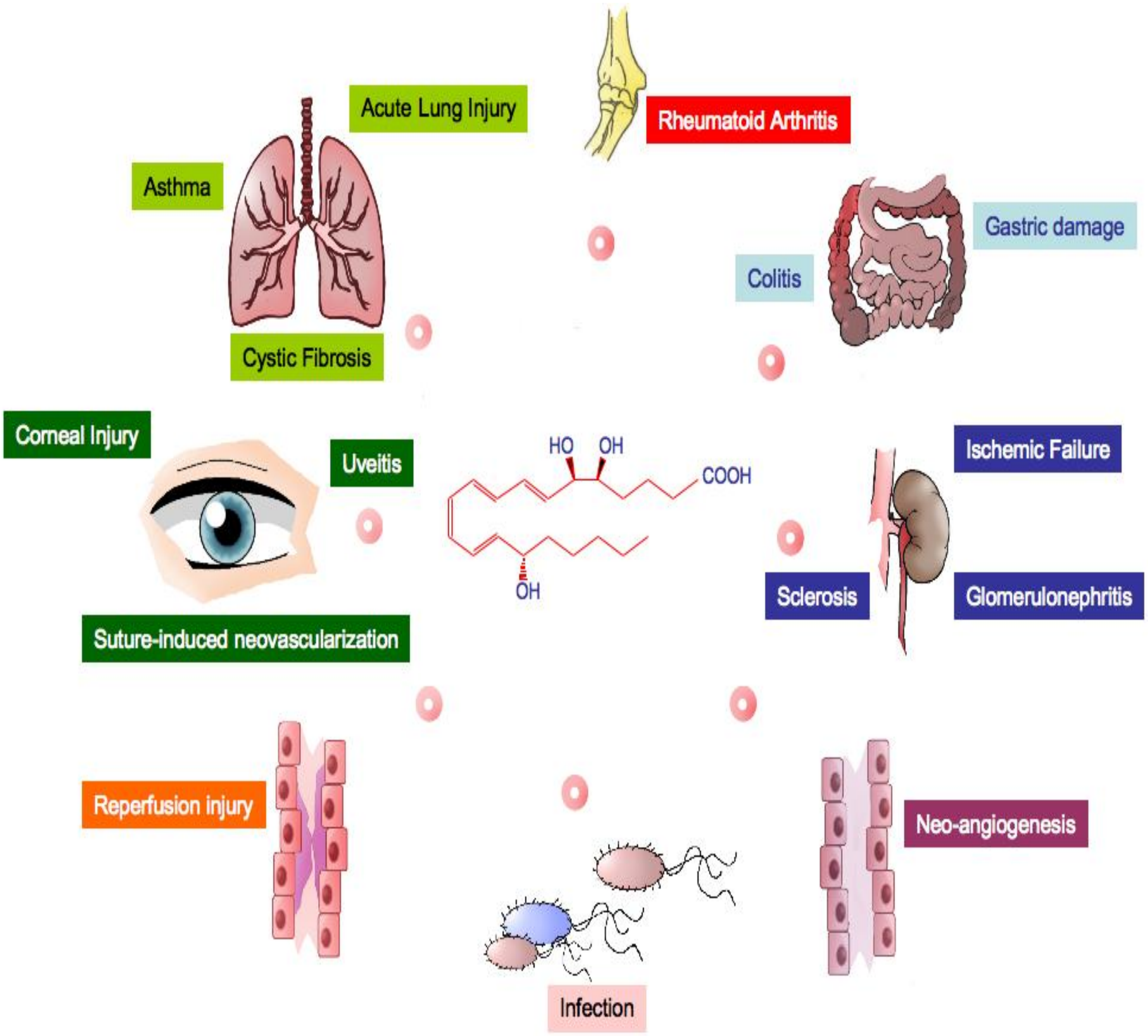

**FIGURE 5.** Clinical settings where administration of lipoxin $A_4$ or ATLa could be beneficial.

### CONCLUSIONS

Evidence that lipoxin and ATL exert potent anti-inflammatory, proresolution bioactions has been consolidated over the years. The bioactions and signaling of these eicosanoids have been largely elucidated, although the list is continuously growing. The significant impact of lipoxin $A_4$, ATLa, and their stable analogs in a large variety of animal studies and *in vitro* models of disease is suggestive of the potential use of these compounds in human therapy. In this respect, in a document dated March 17, 2010, Bayer has announced to investors that lipoxin is in phase I development for inflammatory bowel disease. Additional human studies are now awaited to complete the transition of these eicosanoids from bench to bedside.

### ACKNOWLEDGMENTS

The author wishes to tank Stefano Lattanzio for excellent graphic assistance.
